# Historical review: a short history of German neurology – from its origins to the 1940s

**DOI:** 10.1186/s42466-019-0019-z

**Published:** 2019-04-27

**Authors:** A. Karenberg, H. Fangerau, H. Steinmetz, P. Berlit, M. Grond

**Affiliations:** 1 University of Cologne, Faculty of Medicine and University Hospital Cologne, Institute for the History of Medicine and Medical Ethics, Joseph-Stelzmann-Str. 20, 50931 Cologne, Germany; 2 Institute for the History, Philosophy and Ethics of Medicine, Medical Faculty and University Hospital Duesseldorf, Moorenstr. 5, 40225 Duesseldorf, Germany; 3 Department of Neurology, University Hospital/Goethe University Frankfurt, Schleusenweg 2-16, 60528 Frankfurt am Main, Germany; 4 German Society of Neurology, Reinhardtstr. 27 c, 10117 Berlin, Germany

**Keywords:** Neurology/history, Specialization/history, Germany, National socialism/history

## Abstract

This paper aims at reconstructing the development and role of German neurology between 1840 and 1940. Therefore a couple of original sources as well as selected material form the scattered secondary literature were assessed and reviewed. Since the middle of the nineteenth century, an intricate process of separation from internal medicine and psychiatry gradually led to forming a self-conscious community of German neurologists. While Moritz Heinrich Romberg had constructed a cognitive basis for neurology, scientific founders such as Wilhelm Erb, Carl Wernicke, Alois Alzheimer, Hermann Oppenheim, Max Nonne, and many others established the new discipline within modern medicine. In 1891, the first generation of “pure” neurologists succeeded in founding the German Journal for Neurology (*Deutsche Zeitschrift für Nervenheilkunde*) followed by an autonomous professional organisation, the Society of German Neurologists (*Gesellschaft Deutscher Nervenärzte*) in 1907. A variety of external factors, however, hampered the institutional evolution and thus the implementation of chairs and departments remained quite modest. In 1935, only 2 years after the National Socialists had seized power, the regulatory merger with the psychiatristsʼ society caused the cautious attempts of German neurologists for autonomy to end in complete failure. The imprisonment, murder and expulsion of neuroscientists declared as Jewish or non-Aryan caused profound changes in neurology, medicine, academic life, and health care in general. Further historical research is needed to reconstruct in detail the involvement of German neurologists in racial-hygienic and eugenic research as well as the institutional and scientific development of German neurology after World War II.

## Introduction

Two features characterize the evolution of German neurology. One is its long-lasting relation with internal medicine and psychiatry, the other its late autonomy [14, 17, 24]. In order to fully comprehend this development which is different from many other countries, two watershed moments are of special interest: the foundation of a professional society for neurology in 1907, and the emergence of separate neurological chairs, departments and hospitals from 1955 onwards. As a result, three historical periods can be distinguished:

- The first is epitomized by a unity of neurology and psychiatry in terms of profession, institutions and research methods (ca. 1840–1907).
- The second saw an intensive struggle for disciplinary autonomy by German neurologists (1907-ca. 1955).
- Only during the last period the separation of neurology from psychiatry and internal medicine was realized (ca. 1955 until today).

This paper describes and analyzes the origins of German neurology up to the 1940s. Historical accounts in later issues of this journal will deal in detail with German neurology during the “Third Reich” as well as more recent developments beginning after World War II.

## German neurology in conjunction with internal medicine and psychiatry

Some of the roots of clinical neurology can be traced back to Germany. Moritz Heinrich Romberg, Professor of Internal Medicine at Berlin University, published in 1840 the first volume of his “Manual of the Nervous Diseases of Man”. The final volume appeared in 1846, followed by translations into English, Dutch and Russian. In this work all by then known neurological disorders had been summarized between two covers. Adopting Charles Bell’s and Francois Magendie’s distinction between motor and sensory nerves and spinal root portions, the Berlin clinician was the first to formulate an analytical structural-functional approach towards examining and understanding disease-related dysfunction, mainly of the peripheral and spinal nervous system [23]. Therefore, some authors called Romberg “the first clinical neurologist” [22] and claimed: “Modern neurology begins with Romberg” [11].

Another important starting point for German neurology was the establishment of the first separate wards for neurological patients at Berlin Charité Hospital in 1865 by Wilhelm Griesinger [20] – almost simultaneously with Charcot’s first neurological wards in Paris and the Queen Square Hospital in London. During the early years of German neurology, however, the predominant institution was the joint academic “Psychiatric and Neurological Department” (*Psychiatrische und Nervenklinik*). Community hospitals with neurological wards didn’t exist, and specialized doctor’s offices just began to play a minor role. However, the field was booming, and in 1906 psychiatry (but not neurology) was included in medical board exams.

In the middle of this period a political decision with enormous historical consequences was taken: Against the explicit vote of the Berlin Medical Faculty the Prussian Ministry of Education determined that Griesinger’s “neurological wards” continued to be part of the Psychiatric University Hospital. Thus, autonomy for neurology was refuted. This strategic decision was based on a report drafted by Eduard Hitzig in 1889 [7], well known for his experiments on the cortex. The Hitzig report cannot be overestimated; it perpetuated the institutional unity of both disciplines for another 70 years.

Less contentious was the issue of research strategies. During the decades following Romberg’s pioneering work, the leading paradigm was that of neuropathology. For scholars like Carl Wernicke, the fibre anatomy of the brain was of major interest. Following Broca’s observations on motor aphasia published in 1861, he described sensory aphasia. Wernicke further conceptualized “topic” models of higher-order dysfunction and envisioned that these might hopefully be able to later unify neurology, psychology and psychiatry [23]. Around 1900, however, this paradigm underwent a considerable change: Virchow’s doctrine of cellular pathology was finally transferred to the brain and had a highly productive impact. Some of the most spectacular results of this new morphological research were reported by Alois Alzheimer who was able to distinguish various brain diseases by analyzing tissue alterations, including the disorder that came to bear his name.

Clinical research of the time aimed at defining new disease patterns ranging from muscular dystrophy to Wernicke’s encephalopathy and Alzheimer’s presenile dementia (an eponym invented by Emil Kraepelin [1]). Max Nonne, one of the leading German neurologists of the time, spoke in retrospect of the “Pericleian phase” of his discipline, thus comparing achievements in clinical neurology during the Wilhelminian Empire with the building program in classical Greek history.

When looking for deeper philosophical links one will come across the prevalence of positivistic thinking and, above all, layer models and degeneration theory. Adopted from geology and biology, thinking in layers and hierarchies influenced numerous sciences. The best example from the neurosciences is Hughlings Jackson’s doctrine of higher and lower brain centers, the alteration of which resulted in excitatory and inhibitory symptoms.

**Fig. 1:**
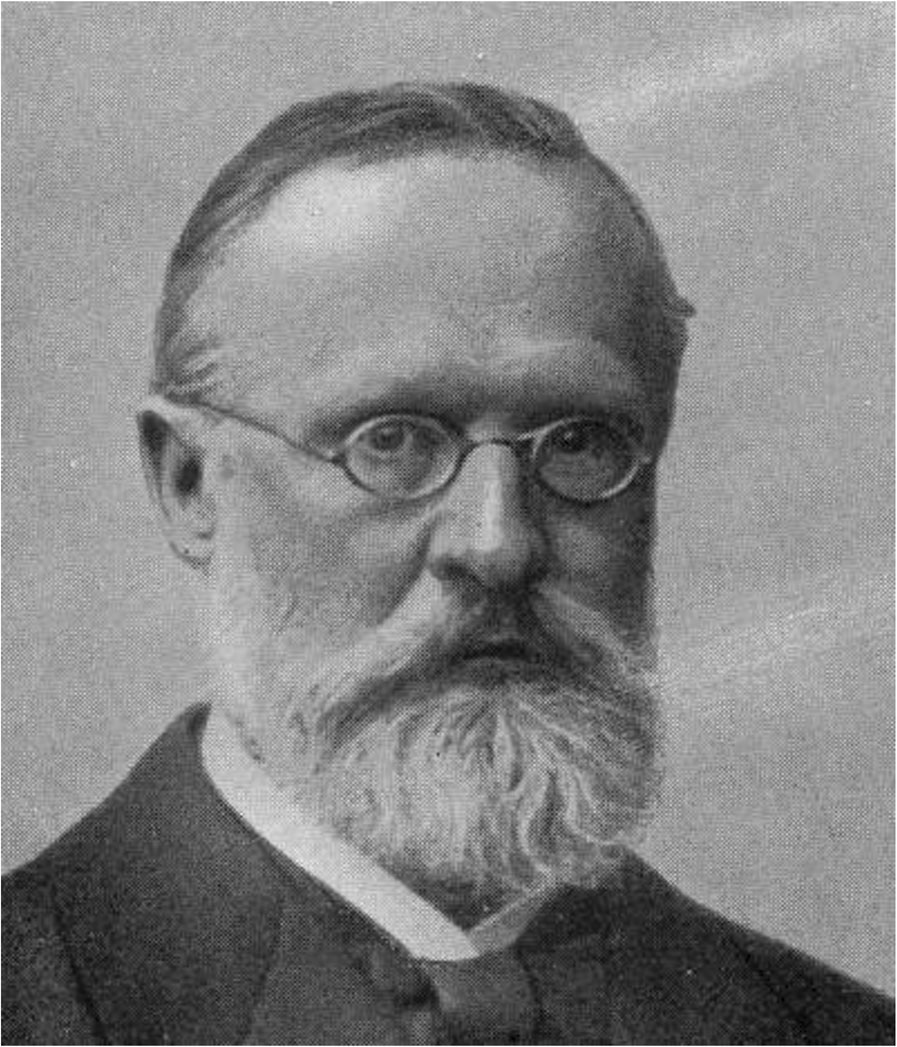
Wilhelm Erb (1840–1921) [Collection Wellcome Images, Creative Commons License DD BY 4.0]

Looking back at this first period, neurology and psychiatry shared a common area of knowledge. Questions of disciplinary autonomy came up only at the end of this period, since neurologically-oriented physicians complained about their “Cinderella status”. One of the spokesmen of the early autonomy movement was Wilhelm Erb, Professor of Internal Medicine at Heidelberg University (Fig. 1). After having gained experience in pathology and internal medicine, and stimulated by teachers such as Nicolaus Friedreich and Amand Duchenne, Erb’s further work was mainly devoted to spinal and muscular disorders, but also to electrotherapy [19, 23]. In fact, he was Germany’s pre-1900 champion of all disorders ranging from the peripheral nerves up to the basal ganglia. In addition, he was among the first who regularly used the reflex hammer, and he clearly stated the need for neurological specialization and education [8]. As Viets has put it: “Perhaps his greatest gift to neurology was in the field of teaching, for it is to Erb we owe the development of an orderly and systematic manner of examination, so fundamental to diagnosis” [25].

**Fig. 2:**
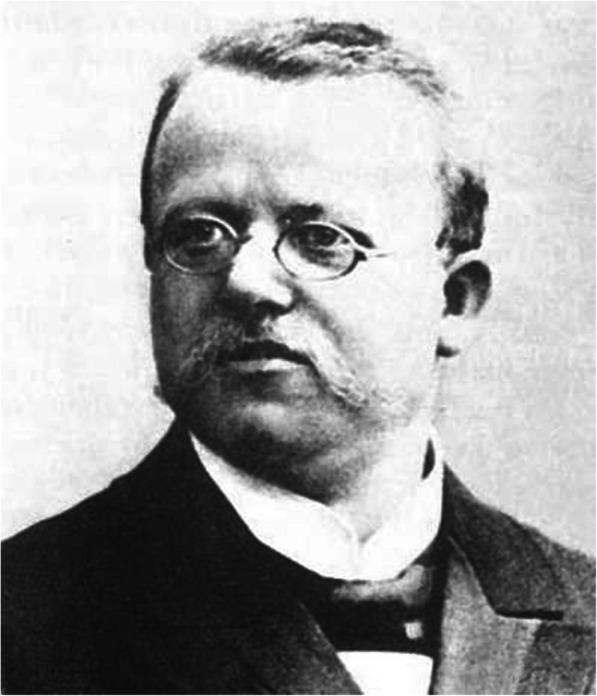
Hermann Oppenheim (1858–1919) [van Gijn J. Hermann Oppenheim (1858–1919). J Neurol 2004;251:1028]

In order to overcome the inferior position of German neurology, Erb propagated along with Hermann Oppenheim (Fig. 2) the foundation of an autonomous “Society of German Neurologists” (*Gesellschaft Deutscher Nervenärzte*) [2, 10]. They succeeded with their plan, and on September 14, 1907, Oppenheim spoke the following words to open the first meeting: “We call ourselves neurologists and are proud to declare ourselves representatives of this science. We are united by the love of the profession”. This “birth certificate” of independent German neurology leads to the second phase of our story: the struggle for power.

## German neurology during the first half of the twentieth century

Around 1900, the scientific panorama started to change. The mechanization of the neurological sector began: spinal tap, radiography of the skull, serology, pneumo-encephalography, angiography, EEG and various other diagnostic procedures mark the beginnings of a technical evolution that is shaping the discipline to date. Neurology grew away from psychiatry, despite common tasks such as the neurosyphilis-issue, despite the fact that some eminent discoveries were made by the sister discipline: The neurologist Max Nonne – later an ardent supporter of “euthanasia” – demonstrated the efficacy of hypnosis in World War I shell-shock sufferers. The psychiatrist Hans Berger developed the EEG, the key diagnostic instrument for the next 50 years: “Indeed, I believe to have found the electroencephalogram of man and hereby publish this for the first time” were the famous words written by 56 year-old Berger in 1929, 5 years after his original recording. Berger’s goal had always been the objective measurement of “psychic energy”. First studies in cats and dogs were followed by direct recordings from the human brain surface during open surgery, and in 1924 from the scalp [23]. Partly due to Berger’s secluded scientific lifestyle, it was not before 1934 that the English physiologist Edgar Adrian recognized the importance of his observations, also lending the name “Berger rhythm” to what Berger had described [4].

In the 1920s and 1930s, three professional archetypes can be identified in Germany [17]: The “pure” neurologist – the most well-known figures being Max Nonne, Heinrich Pette, and, to a certain degree, Otfrid Förster and Viktor von Weizsäcker; the “pure” psychiatrist (for example, Emil Kraepelin and Kurt Schneider); and, the largest group, the “doctors of the nerves”, that is the experts for both mental and nervous disorders. This third group did not work at the university, and instead ran a private or public practice.

**Fig. 3:**
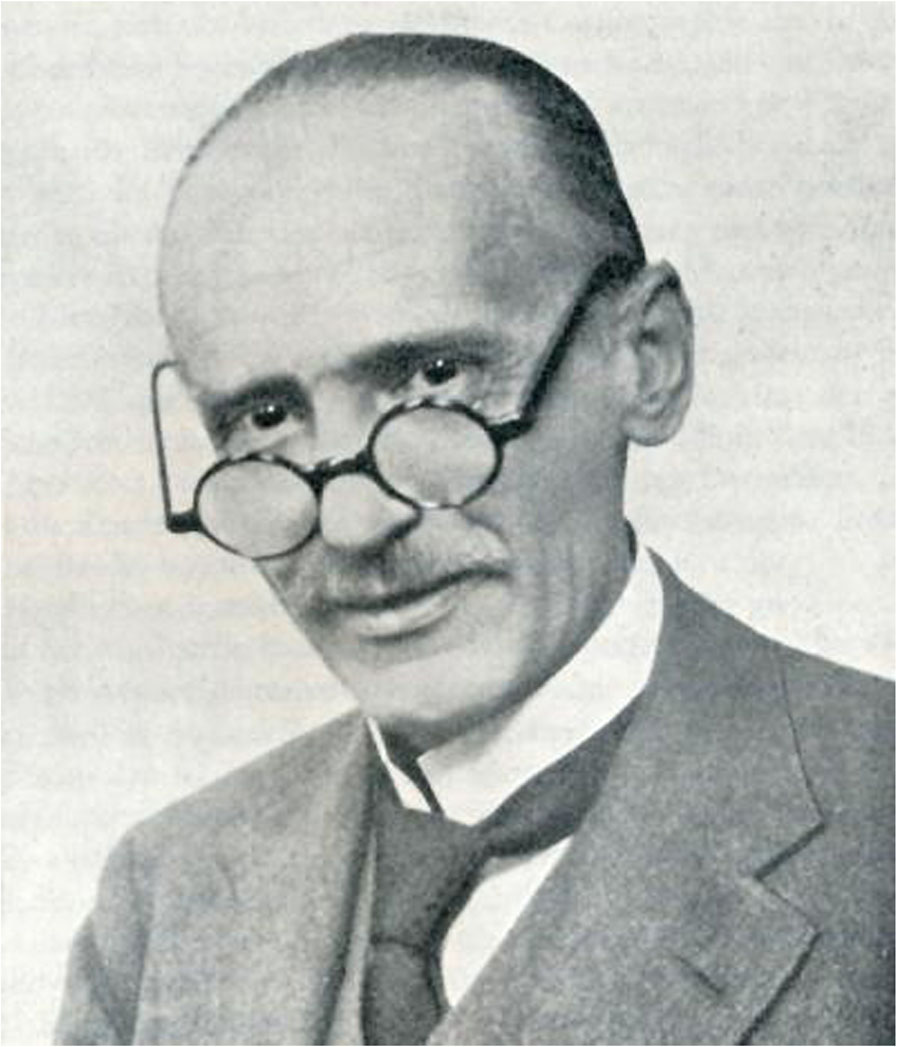
Otfrid Foerster (1873–1941) [Kolle K .Große Nervenärzte, Vol. 2. Stuttgart: Thieme; 1959]

**Fig. 4:**
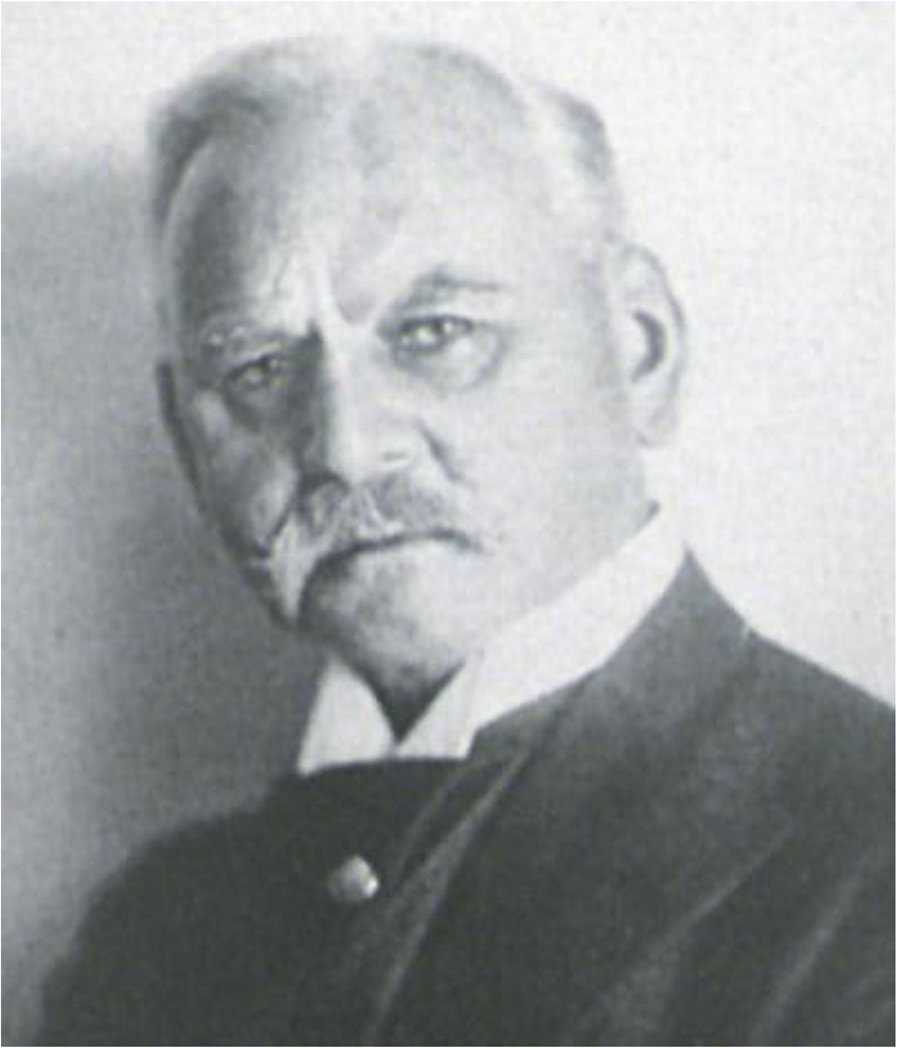
Max Nonne (1861–1959) [Nonne M, editor. Anfang und Ziel meines Lebens: Erinnerungen. Hamburg: Christians; 1971]

Yet at the level of the universities and community hospitals claims for more neurological autonomy found no support. Karl Bonhoeffer, director of the psychiatric and neurological department in Berlin and a prominent figure in the Weimar republic, turned out to be another opponent of the neurologists’ ambitions. He resumed his refusal in three arguments: First, 80% of the neurological ailments had to be ascribed to functional neuroses and thus needed psychiatric expertise; second, diseases of the spinal cord and peripheral lesions couldn’t justify the foundation of new chairs – lectureships and special courses would also do; third, neurology lacked the proper therapeutic means [17]. The alliance of psychiatric and political bigwigs allowed the foundation of only four neurological chairs: in Frankfurt am Main (Ludwig Edinger 1914, but *sans clinique*; [12]), Heidelberg (1919, Johann Hoffmann; [16]), Breslau (Otfrid Foerster [Fig. 3], ca. 1922 who also made innovative contributions to neurosurgery) and Hamburg (Max Nonne (Fig. 4), 1925, with full autonomy; [6]). To put it pointedly in a nutshell: Scientifically, German neurology had grown a giant, institutionally it remained a dwarf [9].

Efforts for autonomy got a deadly blow when the National-Socialists seized power. Their attempts at organizing and controlling medical associations resulted in the forced re-unification of the psychiatric and neurological associations. In 1935 the “German Society for Psychiatry” (*Deutscher Verein für Psychiatrie*) and the “Society of German Neurologists” were incorporated in the newly founded Society of German Neurologists and Psychiatrists (*Gesellschaft deutscher Neurologen und Psychiater*) [21]. But the advent of Nazism in 1933 had more dramatic effects on German neurology than the institutional reorganization. It affected its scientific leaders, quality of clinical care, ethical and scientific standards. For neuroscientists declared as Jewish or non-Aryan, it was the beginning of a distressing history of expulsion from their profession. Some were deported to concentration camps or chose suicide. The long list of emigrants reads like a “Who’s who” of Central European neurology: Josef Gerstmann, Kurt Goldstein, Sir Ludwig Guttmann, Friedrich Heinrich Lewy, Adolf Wallenberg, Robert Wartenberg, and many others [13, 14, 18]. This particularly distressing chapter in the history of German neurology will be the subject of a further project inaugurated by the German Society of Neurology.

German neurologists adjusted their research. Hoping for financial and ideological support many research centers began focusing on hereditary neurological diseases. After the government had passed an eugenic law demanding the forced sterilization of people suffering from allegedly hereditary (neurological and psychiatric) disorders including epilepsy and chorea Huntington, neurological research units for example intensified their efforts in studying the differences between congenital, hereditary and acquired forms of epilepsy. Simultaneously, they offered genetic teaching for SA and SS officials. Additionally, many neurologists served as expert witnesses in Hereditary Health Courts, which decided about the forced sterilization of reported patients. More than 400.000 people were sterilized until 1945.

Even more appalling was the involvement of German brain researchers and neurologists in the ideologically motivated and systematic murder of at least 70.000 mentally ill patients. After 1939, brains from patients who were murdered within the framework of the so-called “euthanasia program”, were made available to researchers. Neurologists wanted to take the chance of participating in this program on the side of research. They used the brains of the murdered patients in order to investigate the neuroanatomy and neuropathology of the killed children and adults. A crucial role played the Kaiser Wilhelm Institut für Hirnforschung in Berlin. Here Julius Hallervorden investigated ca. 700 brains of murdered patients; some of them had been dissected by him personally [3]. Scientific optimism and scientific greed resulted in the dissolution of ethical borders. Next to brain research in the penumbra of the “euthanasia program” neurologists also used the opportunities offered by the NS regime for experimental research neglecting patients’ rights and needs on examining for example Multiple Sclerosis (e.g. Schaltenbrand, see [5, 15]). After the war, although the atrocities committed by neurologists were well known, the neurological community tended to ignore their colleagues’ involvement in the national socialistic crimes. It took until the late 1980s until the community began to discuss and acknowledge its historical responsibility.

## Conclusions

This brief sketch of the development of neurology in Germany can be seen as a plea to the German Society of Neurology and its members to continually reflect their role as physicians, researchers and citizens. Disciplinary autonomy is granted by society in return for the promise of good clinical practice and sound (and ethical) research. Medicine as an experimentally oriented science, however, bears a dangerous potential. In the context of research neurological scientists must not forget the ethical boundaries of their work and must not ignore the principles of non-maleficence and beneficence.

## Abbreviations

DGN: Deutsche Gesellschaft für Neurologie (German Society for Neurology)
EEG: Electroencephalography
NS: National Socialist
SDGGN: Schriftenreihe der Deutschen Gesellschaft für Geschichte der Nervenheilkunde (publication series of the German Society for the History of the Neurosciences and Psychiatry)
